# Seminal Vesiculitis and Prostatitis

**Published:** 1898-04

**Authors:** 


### SEMINAL VESICULITIS AND PROSTATITIS.

Dr. Geo. K. Swinburne (*Jour. Cut. and Gen.-Urin. Dis.*, 1898, CLXXXVI, 119), says that physicians have not recognized the importance of vesiculitis and prostatitis occurring in cases of chronic gonorrhea, and he believes that these conditions will be found in fully thirty-five per cent. of such cases. He then outlines the history and treatment of three cases which had come under his care, and gives the following conclusions:

1. We find a condition of the prostate and seminal vesicles in patients who have never had gonorrhea, which seems to be of a catarrhal nature, which may or may not give rise to symptoms. These symptoms, at present, are apt to be neurasthenic in character; they are benefited by local massage.

2. In chronic urethritis and at the end of prolonged urethritis, or where the posterior urethra has become invaded, the seminal vesicle and prostate should always be examined.

3. Where epididymitis has occurred, seminal vesiculitis is very apt to exist also. This, however, may clear up spontaneously.

4. Tubercular processes should, if possible, be excluded, for massage is apt to render their condition worse.

5. Where live spermatozoa are found by stripping after the urine has stood for some time, it is a good thing that the mucous membrane of the seminal vesicles secretes the proper fluid for preserving the life of the spermatozoa.

6. Stripping the seminal vesicles is a good method to try as a test for sterility, as a test to see whether the ducts between the testis and the seminal vesicle of corresponding side are patent. It may, however, fail.

7. It is necessary to train the finger in making examination for this condition, as in making a vaginal examination.

8. Sometimes at the beginning of treatment nothing or but little material will be expressed. If the treatment is continued, more and more material will be expressed.—*A. M. S. Bulletin.*

